# Narrowed pulse pressure as a predictor of active hemorrhage in hemodynamically stable blunt trauma: Insights from an Asian cohort

**DOI:** 10.12669/pjms.41.12.11505

**Published:** 2025-12

**Authors:** Juan Peng, Dandan Jiang, Shan Liu, Chanjuan Yang

**Affiliations:** 1 Juan Peng, MB., Registered Nurse, Department of Emergency Medicine, The Second Affiliated Hospital, Hengyang Medical School, University of South China, Hengyang, Hunan 421001, China; 2 Dandan Jiang, M.Med., Nursing Officer, Department of Emergency Medicine, The Second Affiliated Hospital, Hengyang Medical School, University of South China, Hengyang, Hunan 421001, China; 3 Shan Liu, M.Med., Attending Physician, Department of Emergency Medicine, The Second Affiliated Hospital, Hengyang Medical School, University of South China, Hengyang, Hunan 421001, China; 4 Chanjuan Yang, M.Med., Attending Physician, Department of Emergency Medicine, The Second Affiliated Hospital, Hengyang Medical School, University of South China, Hengyang, Hunan 421001, China

**Keywords:** Blunt trauma, Pulse pressure, Propensity score matching, Traumatic hemorrhage

## Abstract

**Objective:**

This study aimed to assess the predictive value of pulse pressure (PP) for identifying the critical administration threshold (CAT) in hemodynamically stable patients with active bleeding following blunt trauma.

**Methods:**

This retrospective study included hemodynamically stable blunt trauma patients treated from 2021 to 2022 in our hospital. Patients were grouped by CAT+ or CAT- status. Propensity score matching (PSM) was used to balance baseline characteristics. Logistic regression analyses evaluated the predictive value of PP, while receiver operating characteristic curve (ROC) analysis was performed to determine optimal thresholds in elderly patients (age >60 years).

**Results:**

Of 456 patients, 65 (14.3%) were classified as CAT+. PP ≤40 mmHg was an independent predictor of CAT+ before PSM (OR = 5.931; 95% CI, 2.648–13.284; P < 0.001) and after PSM (adjusted OR = 4.579; 95% CI, 1.193–10.958; P = 0.016). In elderly patients, the optimal PP threshold for CAT+ was ≤41.5 mmHg, with an area under the curve (AUC) of 0.822 (95% CI, 0.724–0.921).

**Conclusion:**

PP is a reliable predictor of critical hemorrhage in hemodynamically stable blunt trauma patients and elderly subgroups, supporting its use in trauma care.

## INTRODUCTION

Traumatic hemorrhage is the leading preventable cause of trauma-related death, accounting for up to 40% of global trauma mortality.^1^,^2^ Delayed recognition and intervention for active bleeding increase mortality and blood transfusion requirements.^3^ Blunt trauma, often caused by high-energy mechanisms, typically involves multiple injuries, complicating early identification of life-threatening bleeding.^4^,^5^ This challenge is amplified in hemodynamically stable blunt trauma patients, where active bleeding often remains undetected during the prehospital phase or initial emergency department (ED) assessment, even with point-of-care ultrasound.^5^,^6^ Traditional tools like the Shock Index (SI), systolic blood pressure (SBP), heart rate (HR), and predictive models are commonly used to assess hemorrhage.^7^–^10^

However, chronic conditions such as atrial fibrillation and hypertension in an aging population compromise the accuracy of these tools.^9^ While predictive models offer greater robustness, their complexity and time requirements limit their practicality in fast-paced ED environments.^11^,^12^ Pulse pressure (PP) has emerged as a simple and effective predictor of trauma-related hemorrhage.^13^–^15^ As a readily measurable parameter, PP remains unaffected by common comorbidities and has shown associations with active hemorrhage. However, most studies have not differentiated between trauma types or have focused exclusively on penetrating trauma, thereby underexploring the unique challenges of blunt trauma. Additionally, many used limited statistical methods that failed to account for confounders, or small sample sizes that reduced model reliability.^16^ This study aimed to evaluate PP’s predictive value as a single parameter for identifying active hemorrhage in hemodynamically stable blunt trauma patients. Propensity score matching (PSM) was utilized to minimize confounding effects and strengthen the reliability of the analysis.

## METHODS

This retrospective study conducted at our hospital during 2021 to 2022.

### Inclusion and Exclusion Criteria:

It included blunt trauma patients presenting from with an initial SBP > 90 mmHg at emergency admission. Exclusion criteria were age < 14 years, pregnancy, transfer to other hospitals, or death in the ED. critical administration threshold (CAT)+ was defined as requiring massive transfusion (MT) (≥10 units packed red blood cells(PRBCs) within 24 hours or ≥3 units per hour) or definitive hemorrhage control. Others were classified as (CAT)-.

### Data collection:

Data included demographics (age, gender, comorbidities, Injury Severity Score (ISS)), ED vital signs, arterial blood gas parameters, and clinical outcomes (transfusion needs, interventions, hospital/ICU stay, survival).

### Ethical Approval:

The study was approved by the ethics committee of the hospital (Ref. No. 2023039; dated March 21, 2023),

### Statistical analysis:

Baseline characteristics were compared before and after PSM using t-tests or Mann-Whitney U tests for continuous variables and chi-square or Fisher’s exact tests for categorical variables. Univariate logistic regression identified variables (P < 0.1) included in a multivariate model. PSM used a 1:1 nearest-neighbor algorithm to balance baseline differences (SMD < 0.1). Post-PSM logistic regression assessed PP’s independent predictive value. receiver operating characteristic curve (ROC) curves and area under the curve (AUC) compared PP thresholds in the elderly subgroup (age > 60 years) and general population. All analyses were two-sided, with P < 0.05 statistically significant, using SPSS (version 26.0, IBM).

## RESULTS

The baseline characteristics before and after PSM are summarized in Table-I. Among 456 patients, 65 (14.3%) were classified as CAT+, and 391 (85.7%) as CAT-. Before PSM, the CAT+ group had lower SBP (median 113.0 mmHg vs. 129.0 mmHg), narrower PP (median 38.0 mmHg vs. 48.0 mmHg), higher lactate (median 2.8 mmol/L vs. 2.3 mmol/L), and lower hemoglobin levels (median 9.7 g/dL vs. 12.3 g/dL) compared to the CAT- group.

**Table-I:** Baseline characteristics before and after propensity score matching.

Characteristics	Before matching (n=456)				After matching (n=96)		
					CAT-	CAT+	p	CAT-		CAT+	p
N	391	65		48		48	
Female sex (%)	103 (26.3)	20 (30.8)	0.553	14 (29.2)		12 (25.0)	0.818
Age(years) (median [IQR])	54.00 [40.00, 65.00]	55.00 [47.00, 65.00]	0.365	51.50 [35.75, 66.00]		53.00 [45.50, 64.25]	0.278
ISS	22.00 [13.00, 29.00]	32.00 [25.00, 39.50]	<0.001*	29.00 [25.00, 34.00]		29.50 [24.00, 38.00]	0.775
Pre-hospital time(min)	173.96 (99.02)	183.69 (80.87)	0.454	186.23 (96.59)		171.25 (78.51)	0.407
Mechanism (%)			0.179				0.783
RTA	205 (52.4)	42 (64.6)		28 (58.3)		30 (62.5)	
Fall	139 (35.5)	18 (27.7)		16 (33.3)		13 (27.1)	
Others	47 (12.0)	5 (7.7)		4 (8.3)		5 (10.4)	
Comorbidities (%)							
Stroke	4 (1.0)	0 (0.0)	0.92	1 (2.1)		0 (0.0)	1
Hypertension	24 (6.1)	7 (10.8)	0.268	4 (8.3)		6 (12.5)	0.738
Diabetes	4 (1.0)	3 (4.6)	0.102	0 (0.0)		1 (2.1)	1
COPD	4 (1.0)	0 (0.0)	0.92	2 (4.2)		0 (0.0)	0.475
CAD	2 (0.5)	1 (1.5)	0.905	0 (0.0)		1 (2.1)	1
Hepatopathy	4 (1.0)	1 (1.5)	1	0(0)		0 (0)	1
ED Vital Sign							
RR(breaths per minute)	20.00 [18.00, 25.00]	21.00 [18.00, 26.25]	0.356	21.00 [17.50, 26.00]		20.50 [18.00, 25.25]	0.783
HR(beats per minute)	87.00 [74.25, 107.00]	93.50 [81.75, 113.00]	0.121	98.50 [79.75, 113.00]		95.00 [81.75, 118.25]	0.927
SBP(mmHg)	129.00 [113.00, 144.50]	113.00 [103.00, 126.00]	<0.001*	112.00 [101.75, 123.00]		111.00 [99.75, 124.00]	0.86
DBP(mmHg)	80.00 [69.00, 91.00]	73.00 [67.00, 86.00]	0.024	68.50 [57.50, 83.50]		69.00 [63.75, 83.25]	0.405
PP(mmHg)	48.00 [42.00, 61.00]	38.00 [32.00, 46.00]	<0.001*	44.50 [39.00, 49.75]		37.50 [32.00, 45.00]	0.01*
PP≤40mmHg(%)	52 (13.3)	38 (58.5)	<0.001*	12 (25.0)		29 (60.4)	0.001*
SPO2(%)	98.00 [95.00, 99.00]	98.00 [93.00, 99.00]	0.247	99.00 [95.00, 100.00]		98.00 [92.75, 100.00]	0.222
Glasgow Coma Scale	15.00 [12.00, 15.00]	15.00 [6.00, 15.00]	0.093	15.00 [6.00, 15.00]		15.00 [12.00, 15.00]	0.554
Laboratory test							
Hb(g/L) (median [IQR])	12.25 [10.10, 13.97]	9.70 [8.70, 11.95]	<0.001*	10.35 [8.90, 12.12]		9.70 [8.85, 12.20]	0.741
BE(mmol/L) (median [IQR])	-3.40 [-6.00, -1.60]	-5.25 [-8.03, -3.35]	0.001*	-6.05 [-7.97, -3.08]		-5.35 [-8.03, -3.30]	0.789
Lac(mmol/L) (median [IQR])	2.30 [1.60, 3.60]	2.80 [2.00, 4.10]	0.008*	2.95 [1.78, 4.88]		2.85 [2.15, 4.23]	0.863
Emergency Intervention(%)							
Massive Transfusion(%)	40 (10.2)	65 (100.0)	<0.001*	13 (27.1)		48 (100.0)	<0.001*
Operation for bleeding(%)	43 (11.0)	61 (93.8)	<0.001*	4 (8.3)		46 (95.8)	<0.001*
Angioembolization for bleeding(%)	19 (4.9)	7 (10.8)	0.107	6 (12.5)		4 (8.3)	0.738
Outcomes							
ICULOS (D) (median [IQR])	5.30 [1.00, 12.50]	5.80 [2.35, 14.25]	0.351	9.00 [3.00, 17.00]		6.05 [2.40, 18.38]	0.427
HLOS(D) (median [IQR])	22.00 [10.00, 40.00]	20.00 [6.00, 41.00]	0.656	23.00 [11.50, 52.00]		26.00 [8.25, 47.25]	0.863
Mortality (%)	37 (9.5)	19 (29.2)	<0.001*	11 (22.9)		14 (29.2)	0.642

***Abbreviations:*** CAT: critical administration threshold; ISS: Injury Severity Score; RTA: Road traffic accident; COPD: chronic obstructive pulmonary disease; CAD: coronary artery disease; ED: Emergency Department; RR: respiratory rate; HR: heart rate; SBP: systolic blood pressure; DBP: diastolic blood pressure; PP: pulse pressure; SPO2:oxygen saturation; Hb: hemoglobin; BE: base excess; Lac: lacticacid; ICULOS: Intensive care unit length of stay; HLOS: hospital length of stay.

After PSM, 96 patients were matched (48 per group), balancing all baseline characteristics except PP, which remained significantly lower in the CAT+ group (median 38.0 mmHg vs. 45.0 mmHg, P < 0.01). Emergency intervention rates were higher in the CAT+ group but excluded from regression analysis to prevent circular reasoning.

### Logistic regression and PSM analysis:

All included variables underwent univariate logistic regression, with those meeting a significance threshold of P < 0.01 reported. Significant predictors included PP ≤40 mmHg (OR = 5.931; 95% CI, 2.648–13.284; P < 0.001), SBP (OR = 0.976; 95% CI, 0.957–0.996; P = 0.001), Hb (OR = 0.874; 95% CI, 0.751–1.016; P = 0.001), BE (OR = 0.988; 95% CI, 0.874–1.115; P = 0.001), and Lac (OR = 1.115; 95% CI, 0.890–1.398; P = 0.013). These variables were included in the multivariate analysis.

In the multivariate model, PP ≤40 mmHg (adjusted OR = 5.931; 95% CI, 2.648–13.284; P < 0.001), SBP (adjusted OR = 0.976; 95% CI, 0.957–0.996; P = 0.018), and ISS (adjusted OR = 1.047; 95% CI, 1.013–1.083; P = 0.007) were identified as independent predictors of CAT+. Detailed regression results are provided in Table-II. After PSM, logistic regression confirmed the independent predictive value of PP≤40mmHg for CAT+ (adjusted OR = 4.579; 95% CI, 1.193–10.958; P = 0.016) Table-III.

**Table-II:** Univariate and multivariate logistic regression analysis for CAT+.

Variables	Primitive cases(n=456)		
	Univariate analysis	Multivariate analysis	
	P	AOR (95%CI)	P
Gender	0.457		
Age(years)	0.266		
ISS	0.001^*^	1.047(1.013-1.083)	0.007^*^
Pre-hospital time(min)	0.453		
Mechanism	0.184		
RR	0.262		
HR	0.330		
SBP	0.001^*^	0.976(0.957-0.996)	0.018^*^
DBP	0.795		
PP≤40mmHg	0.001^*^	5.931(2.648-13.284)	<0.001^*^
SPO2	0.310		
Glasgow Coma Scale	0.182		
Hb	0.001^*^	0.874(0.751-1.016)	0.080
BE	0.001^*^	0.988(0.874-1.115)	0.841
Lac	0.013^*^	1.115(0.890-1.398)	0.344

***Abbreviations:*** CAT: critical administration threshold; ISS: Injury Severity Score; ED: Emergency Department.; RR: respiratory rate; HR: heart rate; SBP: systolic blood pressure; DBP: diastolic blood pressure; PP: pulse pressure; SPO2:oxygen saturation; Hb: hemoglobin; BE: base excess; Lac: lacticacid; AOR: adjusted odds ratio.

**Table-III:** Logistic regression analysis for CAT+ after propensity score matching.

Variables	Univariate analysis (n=96)	
	OR (95%CI)	P
PP≤40mmHg	4.579(1.193-10.958)	0.016^*^

***Abbreviations:*** CAT: critical administration threshold; PP: Pulse pressure; OR: odds ratio

### Subgroup Analysis:

**Fig.1:**
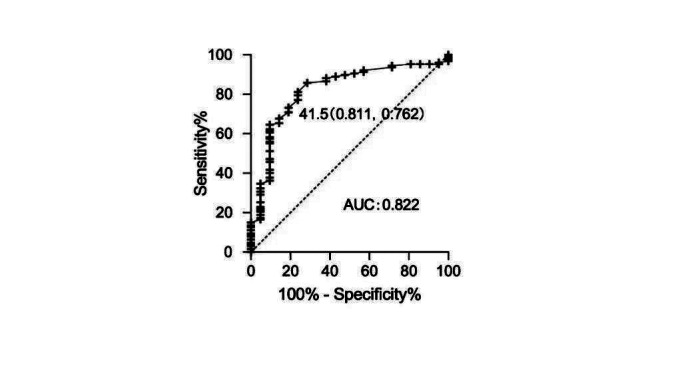
The receiver operating characteristic (ROC) curve for threshold for CAT+ in elder patients.

The ROC curve analysis for the elderly subgroup showed that the optimal threshold for predicting CAT+ was PP ≤41.5 mmHg, with an AUC of 0.822 (95% CI, 0.724–0.921; P = 0.001) (Table-IV and Fig.1). In comparison, PP ≤40 mmHg had an AUC of 0.786 (95% CI, 0.667–0.905; P = 0.001) in this group (Table-IV).

**Table-IV:** The comparison of AUC between the two PP threshold for CAT+ in elder patients.

Threshold	AUC(95%CI)	P
PP≤40mmHg	0.786(0.667-0.905)	0.001^*^
PP≤41.5mmHg	0.822(0.724-0.921)	0.001^*^

***Abbreviations:*** CAT, critical administration threshold; PP, pluse pressure; AUC, area under curve.

## DISCUSSION

In our study, we identified that a PP ≤40 mmHg was an independent predictor of traumatic hemorrhage in patients with blunt trauma requiring MT or definitive hemorrhage control. Notably, this threshold increased in older patients, with the optimal PP threshold for predicting CAT+ found to be ≤41.5 mmHg. These findings confirm that a narrowed pulse pressure is a reliable indicator of active internal bleeding, even in hemodynamically stable blunt trauma patients. The ability of PP to reflect critical physiological changes makes it particularly valuable. In response to acute blood loss, the resulting decline in venous return and cardiac output activates the sympathetic nervous system to preserve adequate tissue perfusion. This response increases peripheral vascular resistance, preserving diastolic pressure while systolic pressure declines due to reduced stroke volume,^17^,^18^ consequently narrowing the PP and signaling hemodynamic instability. This makes PP a practical parameter for identifying active bleeding in trauma patients. To strengthen the reliability of our findings, we employed PSM to control for confounding factors, providing a methodological advantage over simpler statistical approaches.^19^ By balancing baseline characteristics between CAT+ and CAT- groups, PSM ensured more accurate estimates of PP’s predictive value, reinforcing its clinical relevance.

Prior research has established PP as a significant predictor for hemorrhage in trauma patients, especially in informing time-sensitive clinical interventions. In their study, Priestley et al.^14^ reported that a PP falling below 40 mmHg correlated with a greater likelihood of massive transfusion and the necessity for definitive surgical control of bleeding. The study further pointed out that older adults may present with a different hemodynamic profile, necessitating the application of a wider PP threshold, a consideration probably attributable to the physiological alterations accompanying aging. Bankhead-Kendall et al.^14^ further identified narrowed PP as an independent predictor of MT and emergent surgical intervention, although their study was limited to hemodynamically unstable patients. Warren et al.^13^ found that PP <30 mmHg could predict MT and the need for emergent surgical intervention in penetrating trauma. However, our threshold for PP may differ due to variations in injury mechanisms, as penetrating trauma tends to result in more localized and immediate physiological effects, while blunt trauma can lead to more diffuse or delayed hemorrhage, potentially requiring different thresholds for accurate prediction.

However, these findings are less applicable to blunt trauma, where detecting active hemorrhage is more challenging due to subtler clinical presentations. Zhu et al.^20^ combined PP with SI to predict MT, emphasizing the potential of multi-parameter approaches. However, their study did not isolate PP’s independent predictive value and lacked real-world applicability due to methodological limitations. Beyond confirming the clinical relevance of pulse pressure, these prior studies collectively reveal critical knowledge gaps that the present study was specifically designed to address.

Our study fills a significant gap in the literature by focusing on an Asian population, as most previous research has primarily investigated Western cohorts.^13^–^15^,^20^ Given the potential ethnic and physiological differences, our findings offer new insights into the utility of PP for early detection of trauma-related hemorrhage in this specific group. Notably, we identified a PP threshold of ≤41.5 mmHg as optimal for predicting active hemorrhage in elderly patients, reflecting age-related vascular changes like reduced arterial compliance that magnify the hemodynamic impact of narrowed PP. This highlights PP’s adaptability across diverse patient subgroups, supporting its broader role in trauma management.

From a clinical perspective, our findings position pulse pressure as a robust yet straightforward tool. Its ease of measurement makes it highly suitable for the early identification of hemorrhage in trauma settings. By providing early warning signs, PP enables timely interventions—such as transfusions and hemostatic treatments—that can prevent progression to circulatory failure, thereby reducing mortality and complications.^1^,^6^,^21^ Compared to other commonly used indicators, PP offers distinct advantages.^22^-^24^ SBP and tachycardia are often less sensitive in detecting early hemorrhage and can be confounded by factors such as chronic hypertension or beta-blocker use, which obscure physiological responses. Moreover, changes in these parameters typically occur later than PP, delaying critical interventions. Despite its advantages in sensitivity, the utility of the SI can be limited by confounding factors, such as underlying patient comorbidities and certain medications, much like traditional vital signs. Alterations in SI often indicate more advanced physiological compromise, where the prognosis is already poor.^9^,^25^ Furthermore, although multi-parameter models show promise in improving diagnostic accuracy, they often require additional resources like laboratory tests or imaging, which are less feasible in acute trauma settings. In contrast, PP’s simplicity and reliability allow for its immediate integration into trauma care protocols, supporting rapid decision-making and timely interventions that are critical for lifesaving outcomes.

### Limitations:

Firstly, the retrospective design and the fact that it was conducted at a single center may introduce selection bias, which could affect the broader applicability of the results to other populations or healthcare systems. Since the research took place at one institution with a particular patient demographic, the trauma management protocols used there may not be representative of those in other regions or countries. As such, caution should be exercised when extrapolating these results to broader populations or diverse healthcare environments. Second, the absence of prehospital data, particularly prehospital vital signs, restricts our ability to evaluate the utility of PP in the early identification of hemorrhage before hospital arrival. Early detection of hemorrhage in prehospital settings is vital for better patient outcomes. Given the unpredictably rapid progression of acute trauma, effective management demands a commitment to continuous assessment over static data points. A more effective strategy would be to track changes in PP over time, offering a more dynamic and comprehensive understanding of hemorrhage, which could enhance clinical decision-making.

## CONCLUSION

This study established narrowed PP as an effective predictor of active hemorrhage requiring MT and definitive hemostasis in hemodynamically stable blunt trauma patients, with a higher threshold observed in elderly patients. These findings highlight PP’s value as a practical tool for early detection and intervention in trauma-related hemorrhage. Multi-center prospective studies are recommended to validate these results and explore PP’s applicability across diverse trauma populations.

### Future recommendations:

To overcome these limitations, future research should focus on multi-center prospective studies to confirm our findings and evaluate the wider applicability of PP across different populations, healthcare environments, and trauma types. The inclusion of prehospital data in future research would also be invaluable in assessing whether incorporating PP into early triage protocols can enhance early detection and facilitate timely interventions, ultimately improving patient outcomes. Additionally, future efforts should focus on creating predictive tools that leverage dynamic PP profiles to guide and improve clinical decisions in trauma settings.. Continuous monitoring of PP and other vital signs could enable the creation of real-time, adaptive models, allowing clinicians to tailor treatment strategies more accurately based on the evolving clinical status of trauma patients.

### Authors Contribution:

**CY:** Conceived, designed and did statistical analysis & editing of manuscript, is responsible for integrity of research.

**DJ, SL and PJ:** Did data collection and statistical analysis. Critical Review.

**PJ:** Literature search, Did review and manuscript writing.

All authors have read and approved the final version of the manuscript to be published.
